# Reference intervals of routine clinical chemistry parameters among apparently healthy young adults in Amhara National Regional State, Ethiopia

**DOI:** 10.1371/journal.pone.0201782

**Published:** 2018-08-02

**Authors:** Molla Abebe, Mulugeta Melku, Bamlaku Enawgaw, Wubet Birhan, Tekalign Deressa, Betelihem Terefe, Habtamu Wondifraw Baynes

**Affiliations:** 1 Department of Clinical Chemistry, School of Biomedical and Laboratory Sciences, College of Medicine and Health Sciences, University of Gondar, Gondar, Ethiopia; 2 Department of Hematology and Immunohematology, School of Biomedical and Laboratory Sciences, College of Medicine and Health Sciences, University of Gondar, Gondar, Ethiopia; 3 Department of Immunology and Molecular Biology, School of Biomedical and Laboratory Sciences, College of Medicine and Health Sciences, University of Gondar, Gondar, Ethiopia; Inselspital Universitatsspital Bern, SWITZERLAND

## Abstract

**Background:**

Clinical laboratory reference intervals (RIs) are essential for clinical diagnosis, treatment and therapeutic monitoring. Locally established RIs are required to correctly interpret clinical laboratory results. In Ethiopia, clinical laboratory test results are interpreted based on RIs derived from a western population.

**Methods:**

A multicenter cross-sectional study was conducted among blood donors in Amhara National Regional State, Ethiopia from March 2016 to May 2017. A total of 1,175 apparently healthy study participants were included in the study from four blood banks in the region. All clinical chemistry parameters were analyzed using Mindray BS-200E full automated clinical chemistry analyzer. The 95% RIs were estimated using reference limits at 2.5th percentile for the lower reference limit and 97.5th percentile for the upper reference limit. Kolmogorov–Sminorv and Wilcoxon rank-sum tests were used to check data distribution normality and whether partitions were needed between variables, respectively.

**Results:**

RIs established include: ALT 5.13–42.88 U/L for males and 4.3–37 U/L for females; AST 12.13–46.88 for males and 10–43.8 U/L for females; ALP 77.2–475.8 U/L for males and 89–381 U/L for females; amylase 29–309.8 U/L for males and 29–287.9 U/L for females; GGT 7–69.8 U/L for males and 6–39.1 U/L for females; total bilirubin 0.11–1.18 mg/dl for males and 0.08–0.91 mg/dl for females; creatinine 0.48–1.13 mg/dl for males and 0.47–1.09 mg/dl for females; total cholesterol 78.13–211.75 mg/dl for males and 83.6–202.7 mg/dl for females; total protein 5.7–9.7 g/dl for males and 5.6–9.47 for females; triglycerides 36–221.9 mg/dl for males and 35.3–201.5 mg/dl for females; urea 12–43 mg/dl for males and 10–38.7 mg/dl for females; and uric acid 2.7–6.9 mg/dl for males and 2.1–5.9 mg/dl for females.

**Conclusion:**

This study has established RIs for routine clinical chemistry parameters. These RIs are important as they support the interpretation of clinical laboratory results for medical decision making and other health-related activities.

## Introduction

Evidence-based laboratory medicine is an essential part of modern laboratory medicine practices [1]. It is estimated that clinical laboratory data influence 70% of clinical decisions; often providing pivotal information for physicians, nurses, public health officers, and other healthcare providers in the prevention, diagnosis, treatment and management of disease [2]. Technological advancement and economic driving forces have led to major changes in clinical laboratories globally, primarily represented by an increase in testing productivity and efficiency [3].

In the clinical laboratory, reference interval (RI) is the interval between, and including, two reference limits. It is the most widely used medical decision-making tool that separates healthy from diseased individuals. Clinicians compare the values of laboratory reports with the given RIs to make a decision regarding the health status of a given individual in clinical diagnosis, treatment and therapeutic monitoring. Therefore, accurate and reliable RI is an integral part of the process of correct interpretation of clinical laboratory test results for patient care [4–9].

International guidelines recommend that RIs are needed for all tests in the clinical laboratory. Clinical laboratories and diagnostic test manufacturers should establish their own RIs for healthy individuals belonging to a group of homogenous population [10]. However, the majority of clinical laboratories in the world adopt RIs established by manufacturers, rather than developing their own or verifying the applicability of those RIs to their specific population [11]. International organizations also recommend that population-specific clinical laboratory RIs should be established because gender, age, ethnicity, race, diet, geographical location and other factors could affect the physiological value of biochemical parameters [12].

For many African population, the clinical laboratory RIs have not been established, and non-locally derived RIs are usually being used in diagnostic laboratories and clinical trials to screen, diagnose and monitor disease conditions [13]. In Ethiopia, health facilities are dependent on RIs published either in available textbooks or diagnostic kit inserts. Given the dietary habits, geographical location and ethnic diversity as a source of variability, it is necessary to establish population-specific RIs for Ethiopian population. Therefore, the aim of this study was to establish RIs for routine clinical chemistry parameters among healthy adults in Amhara National Regional State, Ethiopia.

## Materials and methods

### Study design, period, and setting

**Fig 1:**
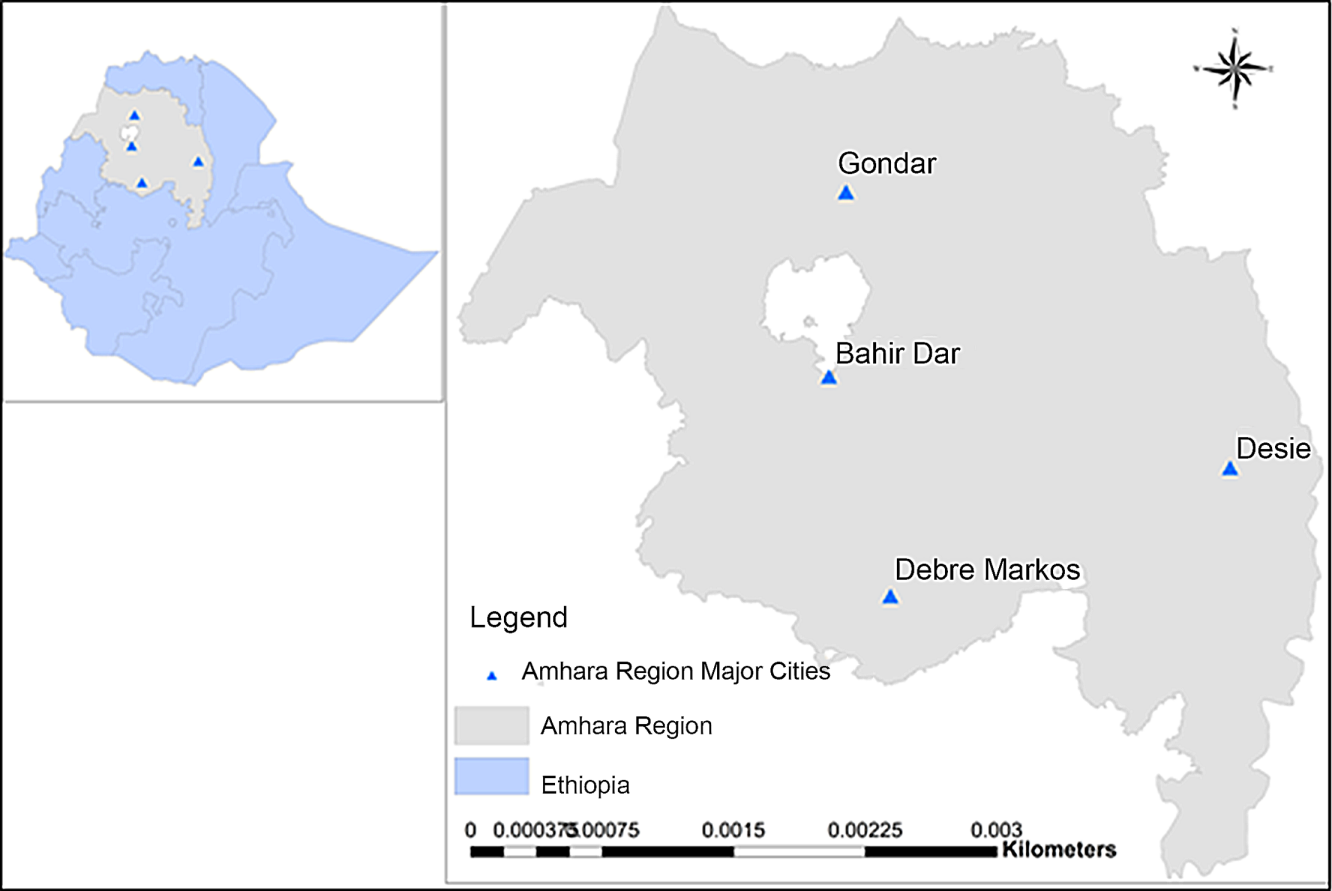
Map of the study area.

A multicenter cross-sectional study was conducted among blood donors in Amhara National Regional State, Ethiopia from March 2016 to May 2017. The Amhara National Regional State (Fig 1) extends from 9° to 13° 45' N and 36° to 40° 30'E. The altitude ranges from 500–4,620 meters above sea level. Based on the 2007 national census report of Central Statistical Agency of Ethiopia, the region has a total population of 17,221,976 (8,641,580 men and 8,580,396 women). The region consists 11 zones, 139 weredas (districts) and 3422 kebeles (neighborhoods) (348 urban and 3074 rural). Amhara (91.5%), Agew-Awi (3.5%), Oromo (2.6%) and Agew Hamyra (1.4%) are the top ranking ethnic groups found in the region. Majority (82.5%) of people living in the region are Orthodox Christians followed by Muslims (17.2%), Protestants (0.2%), Traditional believers (0.03%) and Catholics (0.02%) [14].

Bahir Dar is the capital city of the region, and it is located 560 Kilometers away from Addis Ababa, the capital city of Ethiopia. It has an altitude of 1800 meters above sea level with a total population of 221,991. Debre Markos tow is located 270 Kilometers away from Bahir Dar city. It has an altitude of 2,446 meters above sea level with a total population of 62,497. Gondar town is located 175 Kilometers far from Bahir Dar city. It has an altitude of 2,133 meters above sea level with a total population of 207,044. Dessie town is located 480 Kilometers away from Bahir Dar city. It has an altitude of 2,470 meters above sea level with a total population of 151,174. The study included those participants who were living in and around the aforementioned four areas where ethnic, socio-economic, socio-demographic and altitude differences are not significant among the respective populations [14].

### Reference population

This study included apparently healthy young adults who were selected at each blood donation centre. Disease history questionnaire, physical examination and infectious disease screening tests were used to selected eligible study participants. Adults (age: 18–60 years) with normal body mass index (18–25 kg/m^2^) were candidates of this study. Whereas, adults with known disease like, diabetes mellitus, chronic renal insufficiency, hypertension, ischemic heart disease, anemia, thyroid disease and liver diseases were excluded. Moreover, adults who were taking medications, chronic alcohol abusers, smokers, pregnant and lactating women, positive for transfusion transmissible infections (human immune deficiency virus (HIV), hepatitis B virus (HBV), hepatitis C virus (HCV) and syphilis), and who had a history of jaundice within 3 months and major surgery within 1 year were excluded from the study [10].

### Sample size and sampling technique

The Clinical Laboratory Standards Institute/International Federation for Clinical Chemistry (CLSI/IFCC) recommended non-parametric methods to establish Rls [10]. In order to estimate a non-parametric 95% RI with 95% confidence interval, a minimum of 120 reference individuals are needed per partition (female and male, in our case). From the six blood banks in Amhara National Regional State, four were selected systematically considering their geographical location and population homogeneity. A total of 22 blood donation centres were included in those four blood banks. Then, the reference individuals were recruited by convenient sampling technique at each blood donation centre. More than 120 adults were included per partition because some of them were expected to be excluded by infectious disease screening. A total of 1,290 participants were screened and reviewed at four blood banks of which 1,175 study participants who met the inclusion criteria were selected for this study.

### Data collection and laboratory analysis

Data collectors were trained for three days about the objective of the study, study participants’ rights, confidentiality of patient information, procedure of physical examination, procedure of blood sample collection and measurements, and how to approach and interview participants before the actual data collection. The study participants were contacted when they came to the blood donation centres for donation. Study participants who agreed to give written consent after being informed about the purpose of the study and associated risks were physically examined and interviewed. Socio-demographic data and blood sample were collected from those blood donors who fulfilled the criteria set to say apparently healthy.

About 5 ml of blood sample was collected from each study participant using plane tube. The sample was centrifuged at 2500 rpm (revolution per minute) for 5 minutes to separate serum. Then, the serum sample was tested for HIV, HBV, HCV and Syphilis at each blood bank. Sero-negative serum sample was stored at -20°_C_ until analysis. Finally, the sample was transported to Amhara Public Health Institute (APHI) for analysis. Mindray BS-200E (Shenzhen Mindray Bio-medical electronics co.ltd, China) full automated clinical chemistry analyzer was used for the measurement of biochemical analytes. HUMAN (HUMAN diagnostics, Germany) reagents including control samples and calibrator were used for all clinical chemistry parameters determination.

Clinical chemistry parameters were determined by the methods/techniques described as follows: Alanine aminotransferase and aspartate aminotransferase by kinetic (IFCC without pyridoxalphosphate activation); alkaline phosphatase (ALP) by optimized standard method of DGKC (DEA buffer); gamma-glutamyl transferase (GGT) by L-gamma-glutamyl-3-carboxy-4-nitranilide; amylase by CNP (2-chloro-4-nitrophenol); creatinine by Jaffe (kinetic, without deprotinisation); urea by kinetic urease/GLDH (Glutamate dehydrogenase); uric acid by uricase-PAP (4-aminophenazone); total cholesterol by CHOD-PAP (cholesterol peroxidase- 4-aminophenazone); triglycerides by G-PAP (Glycerol -3-phosphate oxidase- 4-aminophenazone); total protein by biuret; albumin by bromocresol green; and direct bilirubin and total bilirubin by DPD (3,5-dichlorophnyl-diazonium-tetrafloroborate) methods. The viral infectious diseases (HIV, HBV and HCV), and syphilis were screened by enzyme-linked immunosorbent assay (ELISA) and rapid plasma reagin (RPR) techniques, respectively.

### Laboratory data quality management

All pre-analytical, analytical and post-analytical phases of quality assurance cycle were managed based on standard precautions. Each activity including blood sample collection, transportation, storage and analysis was based on good laboratory practices (GLP) using standard operating procedures (SOPs) to ensure data quality. The analysis was done in APHI clinical chemistry laboratory which is closely supervised by Ethiopian Public Health Institute (EPHI). The equipment had been calibrated monthly by type-Autocal. In addition, two levels (normal and pathological) of internal quality control (IQC) samples were run along with the serum sample. The control sample results were interpreted using Westgard multi-rule algorithm. The control sample results had to be within acceptable ranges prior serum sample testing. Moreover, the laboratory had been participating in external quality assessment programs like an onsite evaluation by EPHI and international digital proficiency testing by One World Accuracy three times per year.

### Data analysis and interpretation

Data were cleared, edited, checked for completeness manually and entered to EPI Info version 3.5.3 (CDC, USA) statistical software, and then transferred to SPSS version 20 (IBM, USA) software for analysis. Kolmogorov–Sminorv test was used to check data distribution normality. Wilcoxon rank-sum test was also used to see whether partition was needed between males and females. RIs were calculated in accordance with CLSI/IFCC guideline using non-parametric methods [10]. The 95% RIs were estimated using reference limits at 2.5^th^ percentile for the lower reference limit and 97.5^th^percentile for the upper reference limit.

### Ethical consideration

The study was conducted after ethical approval was obtained from Research and Ethics Institutional Review Board of the University of Gondar. Informed written consent was also obtained from each study participant before the actual data collection. Individuals positive for transfusion-transmissible infections and other disease conditions were linked to nearby government hospitals for further diagnosis and treatment accordingly.

## Results

### Socio-demographic characteristics

A total of 1,175 apparently healthy young adults were recruited to establish the RI of clinical chemistry parameters from four blood banks in Amhara National Regional State, Ethiopia. Out of all study participants, 644 (54.8%) were males. The study participants’ median (IQR) age was 20 (18–22) year. About 706 (60.1%), 977 (83.1%) and 1,075 (91.5%) of the study participants were aged 18–20 years old, students and single, respectively (Table 1).

**Table 1:** Socio-demographic characteristics of blood donors in Amhara National Regional State blood banks, Ethiopia, 2017 (n = 1,175).

Variables		Blood banks				Total
		Debre Markos	Bahir Dar	Gondar	Dessie	
		N %	N %	N %	N %	N %
**Sex**	Male	176(15.0%)	158(13.4%)	144(12.3%)	166(14.1%)	644(54.8%)
	Female	127(10.8%)	135(11.5%	132(11.2%)	137(11.7%)	531(45.2%)
**Age**	18–20	277(23.6%)	196(16.7%)	137(11.7%)	96(8.2%)	706(60.1%)
	21–29	19(1.6%)	92(7.8%)	131(11.1%)	159(13.5%)	401(34.1%)
	30–39	5(0.4%)	5(0.4%)	7(0.6%)	31(2.6%)	48(4.1%)
	40–61	2(0.2%)	0(0.0%)	1(0.1%)	17(1.4%)	20(1.7%)
**Marital status**	Single	293(24.9%)	288(24.5%)	263(22.4%)	231(19.7%)	1075(91.5%)
	Married	10(0.9%)	5(0.4%)	13(1.1%)	72(6.1%)	100(8.5%)
**Occupation**	Student	292(24.9%)	257(21.9)	254(21.6%)	174(14.8%)	977(83.1%)
	Employed	11(0.9%)	34(2.9%)	13(1.1%)	67(5.7%)	125(10.6%)
	Other	0(0.0%)	2(0.2%)	9(0.8%)	62(5.3%)	73(6.2%)
**Total**		303(25.8%)	293(24.9%)	276(23.5%)	303(25.8%)	1175(100%)

**%**: percent, **N**: number.

### Reference intervals of clinical chemistry parameters

We observed statistically significant differences between males and females in clinical chemistry parameters’ RIs. Except total cholesterol, all clinical chemistry parameter values were higher in males than females (Table 2).

**Table 2:** The RIs of clinical chemistry parameters among blood donors in Amhara National Regional State, Ethiopia, 2017 (N = 1,175; Male = 644, and Female = 531).

Analytes	Unit	Sex	Mean	95% CI of Mean	Median	25^th^–75^th^ percentile	2.5^th^–97.5^th^ percentile, RI	p-value*
**ALT/SGPT**	U/L	Combined	15.71	15.22,16.24	14.0	10.0,19.0	5.0–39.0	<0.0001
		Male	17.15	16.50,17.88	15.0	12.0,20.0	5.13–42.88	
		Female	13.97	13.34,14.74	12.0	9.0,16.0	4.3–37.0	
**AST/SGOT**	U/L	Combined	22.84	22.35,23.36	21.0	17.0,27.0	11.0–46.0	<0.001
		Male	24.13	23.46–24.77	23.0	19.0,28.0	12.13–46.88	
		Female	21.27	20.55,22.04	19.0	16.0,24.0	10.0–43.8	
**ALP**	U/L	Combined	199.5	194.13,205.24	174.0	141.75,230.0	87.0–451.28	<0.0001
		Male	217.55	209.09,225.91	186.0	147.0,269.0	77.20–475.8	
		Female	177.56	171.28,184.07	162.0	134.0,204.0	89.0–381.0	
**Amylase**	U/L	Combined	124.84	120.64,128.98	109.0	66.0,172.0	29.0–299.0	<0.0001
		Male	131.07	125.45,136.91	121.5	69.0,179.75	29.0–309.8	
		Female	117.29	111.34,123.32	102.0	61.0,167.0	29.0–287.9	
**GGT**	U/L	Combined	18.39	17.63,19.15	15.0	11.0,21.0	7.0–58.0	<0.0001
		Male	21.08	20.06,22.27	17.0	13.0,24.0	7.0–69.8	
		Female	15.13	14.34,15.97	13.0	10.0,17.0	6.0–39.1	
**Bilirubin (direct)**	mg/dl	Combined	0.22	0.21,0.22	0.19	0.13,0.26	0.02–0.61	<0.0001
		Male	0.25	0.23,0.26	0.21	0.15,0.3	0.04–0.68	
		Female	0.18	0.17,0.19	0.17	0.11,0.23	0.01–0.49	
**Bilirubin (total)**	mg/dl	Combined	0.35	0.34,0.37	0.29	0.21,0.42	0.1–1.1	<0.0001
		Male	0.40	0.38,0.42	0.33	0.24,0.46	0.11–1.18	
		Female	0.30	0.28,0.32	0.25	0.18,0.35	0.08–0.91	
**Total protein**	mg/dl	Combined	7.59	7.52,7.65	7.5	6.8,8.3	5.7–9.6	<0.0001
		Male	7.70	7.61,7.78	7.7	6.9,8.5	5.7–9.7	
		Female	7.45	7.37,7.54	7.4	6.7,8.2	5.6–9.47	
**Albumin**	g/dl	Combined	5.0	4.97,5.04	5.0	4.6,5.4	3.7–6.2	<0.0001
		Male	5.08	5.03,5.13	5.1	4.6,5.5	3.7–6.2	
		Female	4.9	4.86,4.95	5.0	4.5,5.3	3.6–6.1	
**Total cholesterol**	mg/dl	Combined	134.73	132.92,136.56	132.0	113.0,154	80.4–206.6	<0.0001
		Male	132.79	130.39,135.31	130.0	111.0,150.0	78.13–211.75	
		Female	137.08	134.41,139.71	135.0	115.0,159.0	83.6–202.7	
**Triglycerides**	mg/dl	Combined	96.97	94.28,99.80	86.0	61.0,122.0	36.0–215.6	<0.0001
		Male	101.41	97.80,105.00	90.0	63.25,128.75	36.0–221.9	
		Female	91.59	87.61,95.20	80.0	58.0,115.0	35.3–201.5	
**Creatinine**	mg/dl	Combined	0.79	0.78,0.80	0.79	0.7,0.88	0.47–1.12	<0.001
		Male	0.83	0.82,0.84	0.83	0.75,0.92	0.48–1.13	
		Female	0.75	0.74,0.76	0.74	0.66,0.82	0.47–1.09	
**Urea**	mg/dl	Combined	23.35	22.91,23.78	23.0	18.0,28.0	11.0–41.0	<0.0001
		Male	24.53	23.95,25.17	24.0	19.0,29.0	12.0–43.0	
		Female	21.92	21.28,22.51	21.0	17.0,26.0	10.0–38.7	
**Uric acid**	mg/dl	Combined	4.16	4.10,4.23	4.0	3.3,4.9	2.34–6.60	<0.0001
		Male	4.55	4.47,4.63	4.4	3.8,5.2	2.7–6.9	
		Female	3.68	3.60,3.76	3.5	3.1,4.2	2.1–5.9	

*P-value: Wilcoxon rank-sum test for male versus female; **ALT**: Alanine aminotransferase; **ALP**: Alkaline phosphatase; **AST**: Aspartate aminotransferase; **CI**: confidence interval; **dl**: Decilitre; **g**: Gram; **GGT**: Gamma glutamyltransferase; **L**: litre; **mg**: Milligram; **RI**: Reference interval; **SGOT**: Serum glutamate oxaloacetate transaminase; **SGPT**: Serum glutamate pyruvate transaminase; **U**: Unit.

### Confidence intervals for reference limits

The 90% CI of lower and upper reference limits of established RIs of clinical chemistry parameters are calculated and presented in the table below. Table 3 showed that almost all lower reference limits have narrow 90% CI than upper reference limits.

**Table 3:** The 90% CI of reference limits of clinical chemistry parameters RIs among blood donors in Amhara National Regional State, Ethiopia, 2017 (N = 1,175; Male = 644 and Female = 531).

Analytes	Unit	Sex	Range	2.5^th^–97.5^th^ percentile, RI	90% CI (lower reference limit)	90% CI (upper reference limit)
**ALT/SGPT**	U/L	Combined	2.0–62.0	5.0–39.0	4.0,5.0	36.0,45.0
		Male	3.0–60.0	5.13–42.88	5.0,6.0	36.58,48.57
		Female	2.0–62.0	4.3–37.0	4.0,5.0	32.7,42.61
**AST/SGOT**	U/L	Combined	4.0–64.0	11.0–46.0	10.0,11.4	42.2,49.0
		Male	4.0–63.0	12.13–46.88	12.0,13.0	43.0,51.0
		Female	8.0–64.0	10.0–43.8	10.0,11.0	39.0,49.0
**ALP**	U/L	Combined	10.0–583.0	87.0–451.28	74.75,93.0	433.0,474.0
		Male	10.0–583.0	77.2–475.8	53.0,97.72	453.0,495.48
		Female	34.0–575.0	89.0–381.0	80.11,93.8	334.41,403.0
**Amylase**	U/L	Combined	10.0–367.0	29.0–299.0	26.4,32.0	286.0,310.0
		Male	10.0–367.0	29.0–309.8	25.8,32.0	289.55,317.38
		Female	10.0–357.0	29.0–287.9	24.2,33.0	254.0,302.38
**GGT**	U/L	Combined	1.0–96.0	7.0–58.0	6.0,7.0	53.0,67.0
		Male	1.0–96.0	7.0–69.8	7.0,8.0	57.65,76.0
		Female	4.0–89.0	6.0–39.1	6.0,7.0	31.48,49.73
**Bilirubin (direct)**	mg/dl	Combined	0.0–0.85	0.02–0.61	0.01,0.04	0.53,0.66
		Male	0.0–0.85	0.04–0.68	0.02,0.06	0.6,0.71
		Female	0.0–0.85	0.01–0.49	0.0,0.02	0.41,0.59
**Bilirubin (total)**	mg/dl	Combined	0.02–1.89	0.1–1.1	0.08,0.10	0.97,1.18
		Male	0.02–1.89	0.11–1.18	0.09,0.13	1.1,1.28
		Female	0.05–1.39	0.08–0.91	0.73,0.1	0.78,1.04
**Total protein**	mg/dl	Combined	4.4–10.9	5.7–9.6	5.6,5.8	9.5,9.7
		Male	4.4–10.9	5.7–9.7	5.6,5.9	9.6,9.9
		Female	5.1–10.7	5.6–9.47	5.43,5.8	9.21,9.6
**Albumin**	g/dl	Combined	3.0–7.3	3.7–6.2	3.6,3.84	6.1,6.2
		Male	3.0–7.3	3.7–6.2	3.6,3.9	6.1,6.3
		Female	3.1–6.6	3.6–6.1	3.55,3.83	6.0,6.2
**Total cholesterol**	mg/dl	Combined	54.0–242.0	80.4–206.60	77.0,83.0	201.0,212.0
		Male	55.0–242.0	78.13–211.75	74.0,82.0	202.25,215.66
		Female	54.0–235.0	83.6–202.7	78.3,86.0	194.81,210.0
**Triglycerides**	mg/dl	Combined	24.0–255.0	36.0–215.6	35.0,37.4	208.2,221.6
		Male	24.0–255.0	36.0–221.9	33.66,39.0	213.0,233.84
		Female	24.0–250	35.3–201.5	34.48,38.0	190.42,216.07
**Creatinine**	mg/dl	Combined	0.16–1.46	0.47–1.12	0.42,0.50	1.08,1.15
		Male	0.16–1.46	0.48–1.13	0.38,0.55	1.1,1.15
		Female	0.18–1.36	0.47–1.09	0.4,0.51	1.01,1.17
**Urea**	mg/dl	Combined	6.0–56.0	11.0–41.0	10.4,12.0	40.0,43.0
		Male	8.0–56.0	12.0–43.0	12.0,13.0	40.0,44.0
		Female	6.0–50.0	10.0–38.7	10.0,11.0	35.03,41.48
**Uric acid**	mg/dl	Combined	1.7–10.0	2.34–6.60	2.24,2.4	6.5,6.8
		Male	1.8–10.0	2.7–6.9	2.5,2.8	6.66,7.0
		Female	1.7–7.3	2.1–5.9	2.0,2.24	5.5,6.39

**ALT**: Alanine aminotransferase; **ALP**: Alkaline phosphatase; **AST**: Aspartate aminotransferase; **CI**: confidence interval; **dl**: Decilitre; **g**: Gram; **GGT**: Gamma glutamyltransferase; **L**: litre; **mg**: Milligram; **RI**: Reference interval; **SGOT**: Serum glutamate oxaloacetate transaminase; **SGPT**: Serum glutamate pyruvate transaminase; **U**: Unit.

## Discussion

Nowadays, developing countries like Ethiopia are facing double burden of communicable and non-communicable diseases [15]. In this regard, clinical laboratory attempts to play a major role in providing valuable information for prevention, diagnosis and management of life-threatening diseases [2]. Ethiopian population depends on western derived RIs for disease diagnosis and management because of lack of locally established RIs. However, a number of studies showed variations between African and western population derived RIs [16–18]. Thus, it is required to establish local RIs for adequate medical care and related health issues.

As clearly presented in Table 4, the RIs were different for African and western population; specially, the upper reference limits of the majority of clinical chemistry parameters were higher for healthy African population [16, 19–22] than western population [23] (USA and manufacturer represent the western population). In addition, the western study (USA) [23] presented a single RI range for both sexes except uric acid. However, the majority of African based studies [19–22]reported a separate RIs for males and females. These discrepancies are the cause of misclassifications which may have a negative impact in the diagnosis and management of African population. Furthermore, the inconsistency may affect African population in terms of financial resources and time.

**Table 4:** Comparison of clinical chemistry parameter RIs of this study against manufacturer ranges and other similar studies.

Analytes	Sex	Current study	Manufacturer	Northwest Ethiopia [16]	Southwest Ethiopia [19]	Uganda [20]	Tanzania [21]	Ghana [22]	USA [23]
**ALT/SGPT (U/L)**	Combined	5.0–39.0	NA	6.0–43.0	11.0–54.0	6.6–42.8	7.7–48.3	7–51	0–35
	Male	5.13–42.88	0–42	6.0–44.6	11.2–56.0	7.2–43.3	9.1–55.	8–54	NA
	Female	4.3–37.0	0–32	3.0–30.0	10.1–54.0	5.3–39.9	6.7–44.9	6–51	NA
**AST/SGOT (U/L)**	Combined	11.0–46.0	NA	9.0–38.0	12.0–59.0	12.3–34.8	14.3–48.1	14–51	0–35
	Male	12.13–46.88	0–37	10.5–39.0	13.0–59.5	13.2–35.9	15.2–53.4	17–60	NA
	Female	10.0–43.8	0–31	6.0–32.1	12.0–59.9	11.4–28.8	13.5–35.2	13–48	NA
**ALP(U/L)**	Combined	87.0–451.28	NA	52.4–237.0	63.0–376.0	44–151	45.6–158.4	85–241	30–120
	Male	77.2–475.8	80–306	55.3–237.2	55.8–362.9	42–159	45.4–170.4	101–353	NA
	Female	89.0–381.0	64–306	49.0–236.0	70.4–384.4	47–160	45.3–155.0	82–293	NA
**Amylase (U/L)**	Combined	29.0–299.0	0–220	48.0–188.8	NA	45.6–173.6	42.8–164.4	32–139	60–180
	Male	29.0–309.8	NA	45.3–190.0	NA	46–175	49.6–180.1	34–137	NA
	Female	29.0–287.9	NA	48.0–187.9	NA	44–177	41.8–160.4	30–139	NA
**GGT(U/L)**	Combined	7.0–58.0	9–64	NA	NA	8.5–68.5	8.1–107.8	7–61	1–94
	Male	7.0–69.8	NA	NA	NA	8.7–70.7	9.3–120.8	9–71	NA
	Female	6.0–39.1	NA	NA	NA	8.0–41.3	7.3–51.8	6–53	NA
**Bilirubin (direct) (mg/dl)**	Combined	0.02–0.61	0–0.2	0.01–0.80	NA	0.02–0.4	0.04–0.48	0.05–0.23	0.1–0.3
	Male	0.04–0.68	NA	0.02–0.84	NA	0.1–0.5	0.05–0.49	0.05–0.24	NA
	Female	0.01–0.49	NA	0.01–0.71	NA	0.0–0.4	0.04–0.34	0.04–0.22	NA
**Bilirubin (total) (mg/dl)**	Combined	0.1–1.1	0.1–1.2	0.26–2.20	NA	0.4–2.5	0.30–2.40	0.17–1.51	0.3–1.0
	Male	0.11–1.18	NA	0.27–2.20	NA	0.4–2.6	0.35–2.46	0.22–1.87	NA
	Female	0.08–0.91	NA	0.21–2.20	NA	0.3–1.9	0.26–1.83	0.16–1.56	NA
**Total protein (g/dl)**	Combined	5.7–9.6	6.6–8.7	5.3–8.6	4.4–11.6	6.6–8.9	6.6–8.5	5.1–8.7	5.5–8.0
	Male	5.7–9.7	NA	5.3–8.7	4.0–11.4	6.5–8.9	6.7–8.5	4.7–8.6	NA
	Female	5.6–9.47	NA	5.3–8.6	4.6–11.7	6.8–9.0	6.6–8.6	5.5–8.7	NA
**Albumin (g/dl)**	Combined	3.7–6.2	3.8–5.1	NA	NA	3.8–5.3	3.6–5.0	3.3–5.0	3.5–5.5
	Male	3.7–6.2	NA	NA	NA	3.9–5.4	3.7–5.1	3.3–5.0	NA
	Female	3.6–6.1	NA	NA	NA	3.7–5.2	3.6–4.9	3.4–5.0	NA
**Total cholesterol (mg/dl)**	Combined	80.4–206.60	0–190	NA	55.0–276.0	91–233	95.5–213.8	77.3–208.1	<200
	Male	78.13–211.75	NA	NA	52.1–252.2	90–235	89.7–219.3	69.6–193.3	NA
	Female	83.6–202.7	NA	NA	58.0–286.4	100–230	109–212.7	81.2–216.6	NA
**Triglycerides (mg/dl)**	Combined	36.0–215.6	0–150	NA	41.0–264.0	39–281	34.5–255.1	35.4–194.9	<160
	Male	36.0–221.9	NA	NA	41.3–275.8	39–299	34.5–266.6	35.4–194.9	NA
	Female	35.3–201.5	NA	NA	41.0–261.2	34–206	33.7–193.1	35.4–186	NA
**Creatinine (mg/dl)**	Combined	0.47–1.12	NA	0.23–1.22	0.32–1.32	0.5–1.2	0.48–1.02	0.55–1.33	<1.5
	Male	0.48–1.13	0.6–1.1	0.20–1.29	0.3–1.4	0.6–1.2	0.54–1.09	0.63–1.35	NA
	Female	0.47–1.09	0.5–0.9	0.25–1.08	0.3–1.3	0.5–0.9	0.45–0.92	0.53–1.24	NA
**Urea (mg/dl)**	Combined	11.0–41.0	10–50	NA	4.6–35.0	9.9–33.2	9.1–29.5	5.4–34.2	21.4–42.9
	Male	12.0–43.0	NA	NA	4.6–34.5	10.1–33.9	9.3–29.8	5.4–37.2	NA
	Female	10.0–38.7	NA	NA	4.5–35.8	9.4–30.2	8.8–27.4	5.4–32.4	NA
**Uric acid (mg/dl)**	Combined	2.34–6.60	NA	NA	2.0–7.4	3.3–7.8	2.8–7.5	1.5–6.7	NA
	Male	2.7–6.9	3.4–7.0	NA	2.5–7.9	3.5–8.0	3.3–7.7	2.1–7.0	2.5–8.0
	Female	2.1–5.9	2.4–5.7	NA	2.0–7.2	3.0–6.8	2.5–6.1	1.4–6.4	1.5–6.0

**ALT**: Alanine aminotransferase; **ALP**: Alkaline phosphatase; **AST**: Aspartate aminotransferase; **CI**: confidence interval; **dl**: Decilitre; **g**: Gram; **GGT**: Gamma glutamyltransferase; **L**: litre; **mg**: Milligram; **NA**: Not available; **SGOT**: Serum glutamate oxaloacetate transaminase; **SGPT**: Serum glutamate pyruvate transaminase; **USA**: United States of America; **U**: Unit.

This study showed that there is a significant difference of clinical chemistry parameter values by sex. All clinical chemistry parameter values were higher among males than females except for total cholesterol; females had higher total cholesterol value than males. Our study findings are consistent with previous reports of other African countries where there were significant differences for the majority of clinical chemistry parameter values by sex although the higher and lower values were different across studies for both sexes [16, 18, 24, 25].

There is observable clinical chemistry parameter RIs variation between the current study and studies conducted in northwest Ethiopia [16] and southwest Ethiopia [19] (Tables 3 and 4). Except for ALT, there are RIs differences between current study and northwest Ethiopian study. Similarly, the RIs of all clinical chemistry parameters established by southwest Ethiopian study don’t match with our study. These inconsistencies may be due to demographic differences; in the current study, the majority of the study participants were young adults, but the northwest and southwest Ethiopian studies included participants with wider age range (15 to >65 years). Geographic (around 800 Kilometers gap between the current and southwest Ethiopian study areas) and ethnic differences may be accountable for the observed variations. In addition, variablity of analysis methods, equipments and reagents being used may affect the value of clinical chemistry parameters in this different studies [10].

Clinical chemistry parameter RIs are variable among studies conducted in different countries including our study (Tables 3 and 4, [17, 26]). The ALP, amylase, direct bilirubin, albumin and total protein upper reference limits of the current study are higher than studies done in Uganda [20], Tanzania [21], Ghana [22] and USA [23]. On the contrary, the total cholesterol, triglycerides and uric acid upper reference limits of the current study are lower than studies in Uganda, Tanzania and India [20, 21, 27]. In addition, GGT, total bilirubin and total cholesterol upper reference limits of this study are in line with those from Ghana [22]. Ethnic, genetic, demographic, nutritional, cultural, life style, disease distribution, and seasonal differences are important factors which affect the values of clinical laboratory RIs among apparently healthy individuals [27–30]. Furthermore, a number of studies and international guidelines recommended that local clinical laboratory RIs should be established for each homogenous population [10, 31, 32].

This study was limited to young adult blood donors and unable to include children, adolescents and older individuals. Thus, we were not able to confirm whether or not partition among different age groups was needed for clinical chemistry parameter RIs. Dietary pattern of study participants was not assessed because it was difficult to standardize it based on international guidelines. In addition, the resource has limited us to establish the RIs only for routinely requested clinical chemistry parameters in Ethiopian setting.

## Conclusion

The established RIs of clinical chemistry parameters are potentially useful in the diagnosis, management and monitoring of disease progression in the study setting. There are significant RIs variations between the current and other Ethiopian studies. Therefore, further local and nationwide studies, including all age and ethnic groups, are recommended to establish local and national RIs for clinical chemistry parameters.

## Supporting information

S1 File “Data set used for analysis” includes data on reference intervals of clinical chemistry parameters. (XLSX)

## Abbreviations

ALP: Alkaline phosphatase
ALT: Alanine aminotransferase
APHI: Amhara Public Health Institute
CI: Confidence interval
CLSI: Clinical Laboratory Standards Institute
EPHI: Ethiopian Public Health Institute
ELISA: enzyme-linked Immunosorbent assay
GGT: Gamma-glutamyl transferase
GLP: Good laboratory practice
HBV: Hepatitis B virus
HCV: Hepatitis C virus
HIV: Human immune deficiency virus
IFCC: International Federation for Clinical Chemistry
IQC: Internal quality control
RI: Reference interval
RPR: Rapid plasma regain
SGOT: Serum glutamate oxaloacetate transaminase
SGPT: Serum glutamate pyruvate
SOP: Standard operating procedure
